# Respiratory Syncytial Virus NS1 Protein Colocalizes with Mitochondrial Antiviral Signaling Protein MAVS following Infection

**DOI:** 10.1371/journal.pone.0029386

**Published:** 2012-02-27

**Authors:** Sandhya Boyapalle, Terianne Wong, Julio Garay, Michael Teng, Homero San Juan-Vergara, Subhra Mohapatra, Shyam Mohapatra

**Affiliations:** 1 James A Haley Veteran's Administration Hospital, Tampa, Florida, United States of America; 2 University of South Florida Nanomedicine Research Center,Tampa, Florida, United States of America; 3 Department of Internal Medicine, Division of Translational Medicine, Tampa, Florida, United States of America; 4 Department of Allergy and Immunology, Tampa, Florida, United States of America; 5 Department of Molecular Medicine, University of South Florida College of Medicine, Tampa, Florida, United States of America; 6 Departamento de Medicina, Fundación Universidad del Norte, Barranquilla, Colombia; University of Georgia, United States of America

## Abstract

Respiratory syncytial virus (RSV) nonstructural protein 1(NS1) attenuates type-I interferon (IFN) production during RSV infection; however the precise role of RSV NS1 protein in orchestrating the early host-virus interaction during infection is poorly understood. Since NS1 constitutes the first RSV gene transcribed and the production of IFN depends upon RLR (RIG-I-like receptor) signaling, we reasoned that NS1 may interfere with this signaling. Herein, we report that NS1 is localized to mitochondria and binds to mitochondrial antiviral signaling protein (MAVS). Live-cell imaging of rgRSV-infected A549 human epithelial cells showed that RSV replication and transcription occurs in proximity to mitochondria. NS1 localization to mitochondria was directly visualized by confocal microscopy using a cell-permeable chemical probe for His_6_-NS1. Further, NS1 colocalization with MAVS in A549 cells infected with RSV was shown by confocal laser microscopy and immuno-electron microscopy. NS1 protein is present in the mitochondrial fraction and co-immunoprecipitates with MAVS in total cell lysatesof A549 cells transfected with the plasmid pNS1-Flag. By immunoprecipitation with anti-RIG-I antibody, RSV NS1 was shown to associate with MAVS at an early stage of RSV infection, and to disrupt MAVS interaction with RIG-I (retinoic acid inducible gene) and the downstream IFN antiviral and inflammatory response. Together, these results demonstrate that NS1 binds to MAVS and that this binding inhibits the MAVS-RIG-I interaction required for IFN production.

## Introduction

Respiratory syncytial virus (RSV) is a leading etiological agent of lower respiratory tract infections in young children and immunocompromised individuals [1], [2], [3]. RSV infection causes an estimated 64 million cases of respiratory disease and 166,000 deaths annually worldwide and RSV-induced bronchiolitis and pneumonia result in over 2000 deaths and 100,000 hospitalizations annually in the USA [4]. RSV infections recur throughout life since immunity to natural RSV infections fails to generate protective memory responses [5]. No effective vaccine or drug is currently available. Previous efforts to generate a vaccine to RSV using formalin-inactivated virus led to enhanced respiratory disease and the deaths of two vaccinated children after later RSV infection [6], [7]. Subsequently, it was found that formalin-RSV vaccination failed to adequately stimulate receptors of innate immunity, such as Toll-like receptors (TLRs) [8]. New approaches for RSV antivirals involve specifically targeting the RSV proteins that enhance infection and viral survival through RNA interference with the nucleocapsid [9], [10], [11] and nonstructural protein 1 (NS1) [12], [13], [14], [15]. Human RSV is a poor inducer of the type-1 IFN response and infection leads to epithelial damage and activation of inflammatory cytokine signaling. RSV employs multiple defenses against the innate and adaptive antiviral response through impairment of IFN production and interference with lymphocyte activation [16], [17], [18]. RSV nonstructural genes NS1 and NS2, located in the 3′ region of the negative-sense RNA viral genome and transcribed first, are important players early after infection in RSV's subversion of the host antiviral response [12], [19], [20], [21], [22], [23]. We have shown that NS1 plays a very important role in down-regulating IFN production and decreasing activation of IFN-related signaling pathways [12], [13]. However, the precise mechanism remains unknown.

Recently, NS2 was shown to bind to RIG-I but not to MAVS, and was involved in downstream signaling for IFN production [23]. Given the differences in NS1 and NS2 expression and function, we reasoned that investigation of the subcellular localization and interactions of NS1 might shed light on its role in subversion of the innate immune response to RSV infection. Because of a potential role of NS1 in apoptosis [24], we specifically examined the association of NS1 with the mitochondria and mitochondrial antiviral signaling protein (MAVS). We have demonstrated the localization of NS1 to the proximity of mitochondria and the binding of NS1 to MAVS on mitochondria using three microscopy approaches. Our results show that RSV NS1 is associated with mitochondrial MAVS during infection, thus inhibiting MAVS-RIG-I interaction which might indirectly affect IFN production.

## Materials and Methods

### Cell culture

Human embryonic lung fibroblast HEp-2 (CCL-23) cells, human alveolar epithelial A549 (CCL-185) cells and African green monkey kidney Vero (CCL-81) cells were all purchased from the American Type Culture Collection (ATCC) and were cultured in standard Dulbecco's MEM (DMEM) containing 5% heat-inactivated fetal bovine serum, L-glutamine, 100 IU/ml penicillin and 100 µg/ml streptomycin sulfate.

### Viruses

Recombinant RSV strains rA2, rA2ΔNS1, rA2ΔNS2 and rA2-His_6_-NS1 have been previously described [25]. RgRSV, which expresses green fluorescent protein, was a gift from Dr. Mark Peeples. Viruses rA2-His_6_-NS1 and rA2 RSV were grown in HEp-2 cells and harvested when cytopathic effects became visible (2–3 days). RSV-infected cells were subjected to a single round of freeze-thaw cycles and viral supernatant was clarified by centrifugation at 3200 rpmat 4°C for 10 min. Viral titers were obtained through plaque assay with 0.8% methylcellulose overlay and immunostaining with monoclonal murine anti-RSV F antibody, followed by a horseradish peroxidase-conjugated secondary antibody and visualization with 4CN substrate (Kirkegaard and Perry Laboratories). The deletion mutant rA2ΔNS1 was cultured in Vero cells and viral titer was also determined in the same cells. For microscopy studies, A549 and HEp-2 cells were seeded to sub-confluency on glass-bottom culture dishes 24 hrs before infection. On the day of infection, growth media was replaced with Opti-MEM (Invitrogen) containing the appropriate amount of virus suspension for obtaining the indicated multiplicity of infection (MOI).

### Confocal microscopy

A549 cells were infected with rgRSV and imaged from the start of infection for 16 h using a Nikon T inverted microscope running the Perkin Elmer UltraVIEW®VoX 3D Live Cell Imaging System. In a different experiment, A549 cells were infected with rA2 or rA2ΔNS1 and 24 h later the cells were stained with the Image-iT Live Mitochondrial and Nuclear Labeling Kit (Molecular Probes, Invitrogen), using the mitochondrial stain CMXRos, according to the manufacturer's protocol. Cells were fixed with 4% paraformaldehyde, permeabilized with cold 0.2% Triton X-100 in PBS for 10 min, washed twice with PBS and incubated with primary rabbit IgG antibody conjugated to ZenonAlexa Fluor (Invitrogen). Alexa 488 was conjugated to anti-His_6_-NS1antibody and Alexa 647 was conjugated to anti-MAVS antibody (Bethyl Laboratories). Stained cells were viewed under a Leica TCS SP2 laser scanning confocal microscope.

### Fluorescent imaging of histidine-tagged protein using cell permeable Ni^2+^NTA-BM dye in live cells

HEp-2 cells were seeded in 35 mm glass-bottom confocal culture dishes and infected with rA2-His_6_-NS1 at an MOI of 2 for 10 h. Ni^2+^-NTA_2_-dibromobimane (Ni^2+^-NTA_2_-BM) was prepared as previously described [26], [27] and used to label rA2-His_6_-NS1-infected live HEp-2 cells 10 hrs post-infection. 50 µM NTA_2_-BM was complexed with a 10-fold molar excess of NiSO_4_ and incubated with ammonium bicarbonate buffer-washed RSV-infected cells, together with a mitochondrial labeling probe, CMXRos and nuclear (DRAQ5, Cell Signaling) stain, following manufacturers' instructions. Infection with rA2 was used as a control to test target specificity. Fluorescence was visualized by laser confocal microscopy at wavelengths of 405, 563 and 633 nm. Cells were also photographed using differential interference contrast (DIC). A series of 0.5 µm Z-stack images was collected of each specimen and experiments were performed in triplicate. Semi-quantitative analysis with the JACoP provided Pearson's and Manders' overlap coefficients (http://rsb.info.nih.gov/ij/plugins/track/jacop.html).

### Immunogold electron microscopy

A549 cells were grown to near confluence in T-75 flasks (USA Scientific), infected with 0.5 MOI of rA2-His_6_-NS1 and harvested at 12 and 24 h post-infection. The cells were pelleted and fixed in 1% paraformaldehyde, 0.5% glutaraldehyde, 0.05% sodium cacodylate, pH 7.1, for 10 min at 4°C, then in 2% paraformaldehyde, 2.5% glutaraldehyde, 0.05% sodium cacodylate, pH 7.1, for 30 min at 4°C. After washing three times (10 min each time) with 0.05 M sodium cacodylate, the cells were dehydrated with a series of ethanol concentrations (50%, 70%, 85%, 95%, and 3× with 100%) for 30 min for each step at 4°C. Cells were then infiltrated with ethanol∶LR White Resin using ratios of 1∶1 (2 h at 4°C), and 1∶3 (overnight at 4°C) followed by pure LR White (24 h at 4°C, with a resin change after 8 h). The cells were then embedded in gelatin capsules, and resin was polymerized at 4°C for 48 h under UV light. The blocks were sectioned at the Lisa Muma Weitz Advanced Microscopy & Cell Imaging Core Laboratory (University of South Florida) and the sections were laid on nickel grids. Gold labeling of the nickel grids was carried out as described previously [28]. Grids were treated with 25 µl of TBS-supplemented buffer (0.05 M Tris, 0.85% NaCl, pH 8.3–8.5, 0.5% normal goat serum, 0.5% normal pig serum and 0.5% BSA) with 3% nonfat dry milk for 2 h at room temperature. Grids with thin sections were incubated with 50 µl of anti-MAVS rabbit antibody (1∶100 dilution) diluted in TBS-supplemented buffer with 3% nonfat dry milk for 3 h at room temperature, washed and stained with the anti-rabbit antibody conjugated with 15 nm gold (Ted Pella, Inc.) for 1 h at room temperature. The grids were washed thoroughly and treated with 1∶20 dilution of 5 nm gold-Ni-NTA (Molecular Probes) that binds to His_6_ tags.The grids were washed three times with TBS buffer, and incubated with 25 µl of 1∶100 dilution of goat anti-rabbit antibody conjugated with 10 nm gold particles (Ted Pella Inc.) for 1 h at room temperature. After stream washing and three washes with distilled water (10 min each), the grids were dried and stained with 2% aqueous uranyl acetate for 5 min and examined on a JOEL 1200 EX scanning/transmission electron microscope at 80 kV.

### Plasmids

The plasmids used in the study were pNS1-Flag, pFlag vector control for the mitochondrial fraction isolation, pVAX, pNS1, pRIG-I (Invivogen) and pMAVS (Invivogen). Cells were transfected with the specified amounts of DNA using Lipofectamine 2000 (Invitrogen). Plasmid pNS1 was constructed by subcloning a codon-optimized RSVNS1 sequence with restriction enzyme digestion into the parental pVAX vector. pNS1-Flag was similarly constructed by restriction enzyme digest into pFlag vector, with Flag on the amino terminus of NS1. Constructs were verified with sequencing and molecular weight of pNS1-Flag was ∼17 kDa when probed with an anti-Flag antibody.

### Mitochondrial isolation

20×10^6^ cells were either transfected with pNS1-Flag or pFlag vector alone, then the mitochondrial fraction was isolated using a mitochondrial isolation kit (ThermoFisher Scientific). The mitochondrial protein was estimated using the BCA protein assay (ThermoFisher Scientific) and subjected to western blot analysis as described below.

### Immunoprecipitation

30×10^6^ cells were either transfected with the indicated plasmids or infected with RSV at the specified MOI. At specified times, the cells were lysed, and 500 µg aliquots of each protein sample were incubated with the indicated antibody and immunoprecipitated using protein A-agarose beads (Invitrogen). Immunoprecipitation (protocols from eBioScience) was performed using anti-Flag antibody (Sigma), anti-MAVS antibody (Bethyl Laboratories), or anti-RIG-I (Cell Signaling) antibodies and the antigen-antibody complexes were evaluated by western blot analysis.

### Western blot analysis of proteins

Aliquots of the mitochondrial proteins (100 µg) or the immunoprecipitated complexes were boiled for 5 min in 2× protein denaturation buffer (2.3% SDS, 10% glycerol, 5% 2-mercaptoethanol, 62.5 mMTris-HCl and 0.01% bromophenol blue, pH 6.8) and were resolved by electrophoresis on 4–20% SDS-PAGE gels (Bio-Rad Laboratories). Proteins were transferred for 30 min with Towbin buffer (10 mMTris base, 96 mM glycine in 10% methanol) to nitrocellulose membranes (Bio-Rad). Blots were blocked for 30 min at room temperature with 5% nonfat dry milk and 0.1% Tween-20 in Tris-buffered saline (TBS). Blots were separately probed with rabbit anti-Flag antibody (Sigma), mouse anti-MAVS (Santa Cruz), anti-COX-IV (Cell Signaling), anti-RIG-I antibody (Cell Signaling) or polyclonal rabbit antiserum against recombinant His_6_-NS1 [25], and finally, the protein bands were detected by ECL (ThermoFischer Scientific, SuperSignal West Pico).

## Results

### Colocalization of RSV NS1 with mitochondria of infected cells

**Figure 1:**
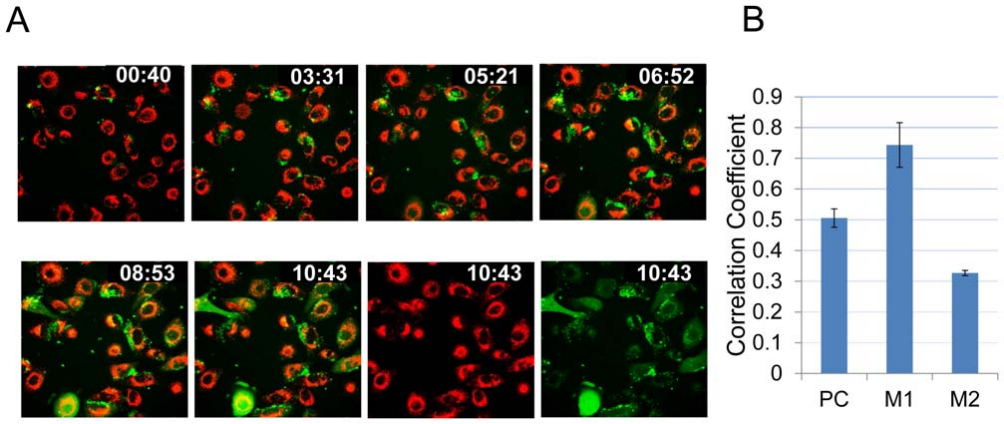
Live cell imaging of Rg-RSV-infected cells. (**A**). A549 cells were seeded in 35 mm glass-bottom fluorodishes (Fisher Scientific) and stained with CMXRos, live mitochondrial labeling kit (Molecular Probes) for 15 min at 37°C, washed and then fresh medium (DMEM with 2% FBS) was added to the cells. Cells were infected with rgRSV at MOI of 1 and visualized on a Perkin Elmer UltraVIEW®VoX 3D Live Cell Imaging System equipped with a Nikon T inverted microscope. The cells were maintained at 37°C with 5% CO_2_ throughout the imaging (16 h post-infection). Images were taken at 0∶40, 3∶31, 5∶21, 6∶52, 8∶53, and 10∶43 hours post-infection. The last two images in the bottom panel show mitochondrial staining and GFP alone respectively at 10∶43 h p.i. The colocalization of GFP (green) with mitochondria (red) is shown as yellow. (**B**). The Pearson's and Manders' overlap coefficients are represented as the average of ten individual cells at 10∶43 hp.i.

In an effort to track the very early events of RSV infection, we used 4-D live cell imaging (Perkin Elmer UltraVIEW®VoX 3D Live Cell Imaging System) of A549 cells stained with live mitochondrial stain CMXRos (red in Figure 1) and infected with rgRSV at 1 MOI. Figure 1Ashows images at several time points (0∶40, 3∶31, 5∶21, 6∶51, 8∶53, and 10∶43 h) after infection. The last two images of the bottom panel showmitochondrial staining (red)and GFP (green)at 10∶43 h p.i. The colocalization of GFP (green) with mitochondria (red) is shown as yellow (merged). The Pearson's and Manders' overlap coefficients were derived with the JACoPImageJ tool (http://rsb.info.nih.gov/ij/plugins/track/jacop.html) and are the average of ten individual cells at 10∶43 h p.i.(Fig. 1B). M1 signifies the correlation of mitochondria overlapping GFP, while M2 indicates the overlap coefficient of GFP to mitochondria.

**Figure 2:**
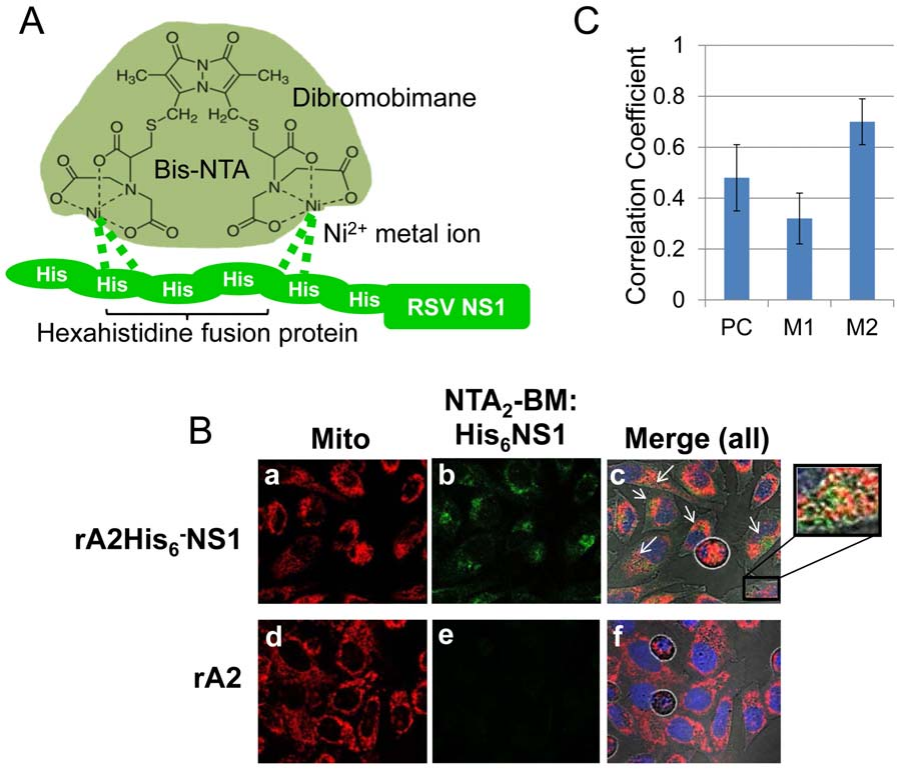
Co-localization of His_6_-NS1 with mitochondria in infected cells using Ni^2+^-NTA_2_-BM. (**A**) Structure of Ni^2+^-NTA_2_-BM [26]. (**B**) rA2-His_6_-NS1 or rA2 were used to infect HEp-2 cells at a MOI of 2. At 10h p.i., infected cells were labeled with CMXRos (red), DRAQ5 (blue), and 50 μMNi^2+^-NTA_2_-BM. Fluorescence was detected using an Olympus FV1000 MPE multiphoton laser scanning microscope. The cells were maintained at 37°C with 5% CO_2_ throughout the imaging (Z min). Shown are micrographs of rA2-His_6_-NS1-infected cells collected 10 h p.i (*a–c*)and rA2 RSV at 10 hp.i. (*d–f*) demonstrating site-specificity for Ni^2+^-NTA_2_-BM. Excitation wavelengths of 405, 563 and 633 nm and differential interference contrast (DIC) were used to collect a series of 0.5 µm Z-stack images for each specimen and experiments were performed in triplicate. Images *a* and *d* show the mitochondria (red), *b* and *e* represent His_6_-NS1 (green) and *c*and*f* are the merged images of red and green along with the blue nuclear stain DAPI and DIC. (**C**). Thresholds for the Z-stack images collected in (**B**) were acquired using RenyiEntropy AutoThreshold ImageJ plugin (Landini) and Pearson's and Mander's coefficients derived with JACoP plugin (Bolte & Cordelieres). The Mander's coefficient M2 indicates that ∼70% of the His_6_-NS1 detected overlaps with CMXRos-stained mitochondria at 10 hrs p.i. in rA2-His_6_-NS1-infected cells.

To investigate binding of NS1 to mitochondria in living cells, we used Ni^2+^-NTA_2_-dibromobimane conjugates (Ni^2+^-NTA_2_-BM) as described previously, which exploits the interaction between polyhistidine and metal ion NTA compounds [26], [29]. The advantage of the chemical labeling method is that it does not require fixation or permeabilization of the cells with their potential for introducing artifacts. The chemical structure of Ni^2+^-NTA_2_-BM and its interaction with the hexahistidine sequences fused to NS1 is illustrated in Figure 2A. This dye was used to detect the His_6_-tagged NS1 expressed by rA2His_6_-NS1 in infected HEp-2 cells. rA2, which expresses wild-type NS1, was used as a control.Merged (or individual channels for detecting the mitochondria and Ni^2+^-NTA_2_-BM-bound His_6_-NS1 are shown in Figure 2B, with BM in green and mitochondrial probe in red (Fig. 2B). Differential interference contrast (DIC) was used to monitor cell morphologyand nuclear staining is shown in blue. Labeling of NS1 occurred in rA2-His_6_-NS1-infected cells (top panel, *a–c*), but not in cells infected with rA2 encoding wild type NS1 (bottom panel, *d–f*) at 10 h p.i. demonstrating that Ni^2+^-NTA_2_-BM is membrane permeable and specific for the His_6_ tag. Overlap of CMXRos and labeling of His_6_-NS1 peaked at 10 h and several colocalization points are shown as white arrows in the merged image (Fig. 2B/c).The inset provided in image *c*shows the higher magnification of the final merged image (mito-NTA_2_-BM). Z-stack images of RSV-infected HEp-2 were collected from four independent experiments. The Pearson's and Manders' overlap coefficients were derived with the JACoPImageJ tool (http://rsb.info.nih.gov/ij/plugins/track/jacop.html) and are the average of ten individual cells (Fig. 2C). M1 signifies the correlation of mitochondria overlapping His_6_-NS1, while M2 indicates the overlap coefficient of His_6_-NS1 to mitochondria. These results show that His-NS1 is localizedwith mitochondria in rA2His_6_-NS1-infected cells.

### Colocalization of RSV NS1 with MAVS by immunogold electron microscopy

**Figure 3:**
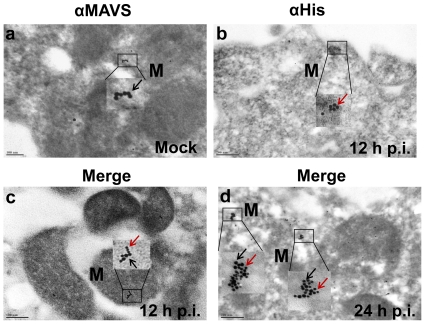
Colocalization of NS1 with MAVS by using immunogold electron microscopy. A549 cells were mock-infected or infected with 0.5 MOI rA2-His_6_-NS1 for 1 h. Cells were harvested at 12 and 24 h post-infection and processed for immunogoldelectron microscopy. Thin sections on nickel grids were treated with rabbit anti-MAVS antibody (1∶100 dilution) for 3 h at room temperature, washed and then stained with 15 nm gold-labeled anti-rabbit secondary antibody. The grids were washed thoroughly and treated with a 1∶20 dilution of 5 nm gold-Ni-NTA (Molecular Probes) that binds to His tags. Cells infected with rA2-His_6_-NS1 were stained for His-NS1 with 5 nm gold (red arrow) and for MAVS protein on mitochondria with 15 nm gold (black arrows). (*a*) Ultrathin sections of uninfected cells showing the 15 nm gold staining of MAVS protein (black arrow). (*b*) Ultrathin sections of infected cells 12 h p.i. showing His_6_ protein stained using 5 nm gold-Ni-NTA. The inset shows His_6_-NS1 localized on the mitochondria. (*c & d*) Sections of cells 12 and 24 h p.i. showing colocalization of NS1 with the MAVS protein. Insets (1 & 2) show colocalization of NS1 (red arrow) and MAVS (black arrow).

Colocalization of RSV NS1 with MAVS was independently validated using immunoelectron microscopy.A549 cells were infected with 0.5 MOI rA2His_6_-NS1 for 1 h, and cells were harvested at 12 and 24 h post-infection. Figure 3a shows MAVS protein in sections of uninfected cells stained with rabbit anti-MAVS antibody followed by secondary anti-rabbit antibody conjugated with 15 nm gold (black arrow). Figure 3b shows His_6_-NS1 in sections of cells stained with 5 nm gold-Ni-NTA 12 hours after rA2-His_6_-NS1 infection (red arrow). Figure 3c and d show cell sections 12 and 24 h post-infection respectively, stained with anti-MAVS antibody (15 nm gold) followed by Ni-NTA (5 nm gold) antibodies. These results demonstrate that His_6_-NS1 colocalizes with MAVS on mitochondria (M).

### RSV NS1 colocalizes with MAVS independent of RSV NS2 during infection

**Figure 4:**
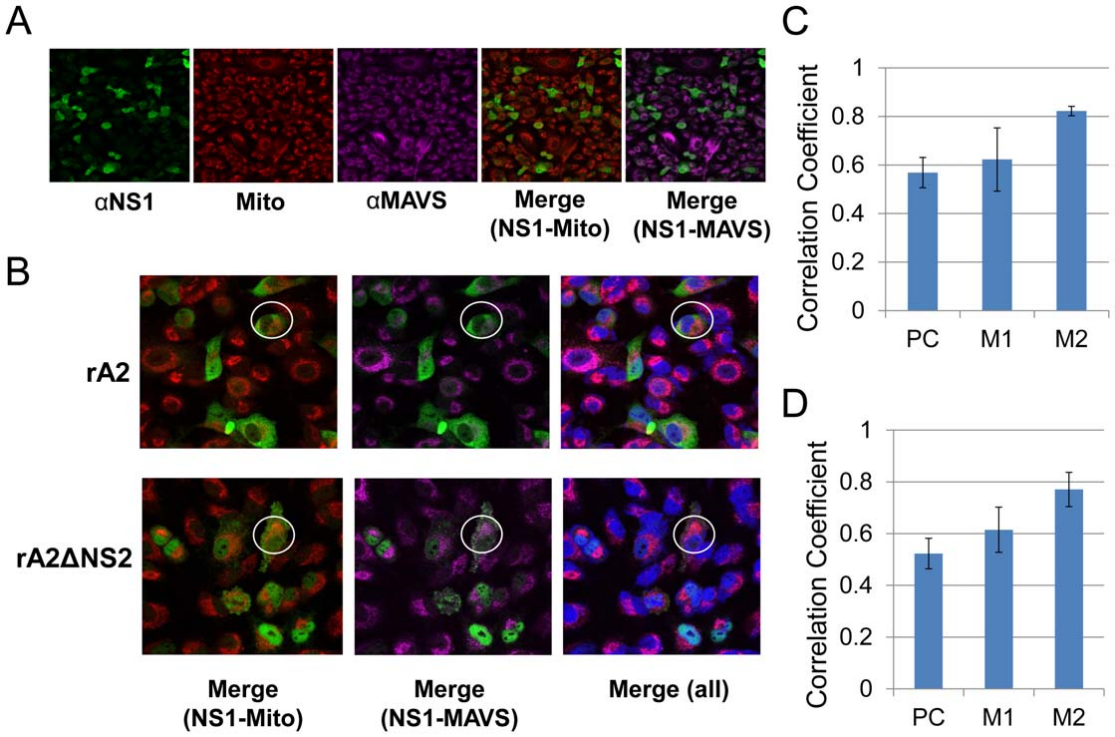
Co-localization of NS1 with MAVS in mitochondria during RSV infection. A549 cells were infected with rA2 or rA2ΔNS2 (0.1 MOI).Twelve hours after infection, cells were stained with the mitochondrial stain CMXRos (red). Cells were then fixed and stained with Alexa 488-conjugated anti-NS1 antibody (green) and Alexa 647-conjugated anti-MAVS antibody (purple). Stained cells were viewed under the Leica TCS SP2 laser scanning confocal microscope (**A**) Images (400× magnification) show individual staining colors, and merged images of NS1-mitochondria and NS1-MAVS are also shown. (**B**) The images are a 5× enlargement of a 400× image. The top panel shows the cells infected with rA2 and the bottom panel the cells infected with rA2ΔNS2. The areas marked by white circles show the presence of NS1 in mitochondria and colocalization with MAVS. The first pair of images are merges of NS1 and mitochondria. The second pair show NS1 merged with MAVS and the last pair shows all three. (**C**). Correlation analysis of the co-localization of NS1 with mitochondria in rA2 infected cells. (**D**). Correlation analysis of the colocalization of NS1 with mitochondria in rA2ΔNS2 infected cells.Pearson's coefficient for MAVS and NS1 is shown along with Manders' coefficients (M1 & M2), which represent the fraction of the mitochondrial red overlapping with NS1 green and the fraction of NS1 green overlapping with the mitochondrial red, respectively.

To determine whether RSV NS2 is involved in NS1-MAVS interaction, A549 cells were infected with rA2 or rA2ΔNS2 (0.1MOI) and at 12 h post-infection (p.i.), cells were stained with the mitochondrial stain CMXRos (red), Alexa 488-conjugated anti-His_6_-NS1(green), and Alexa 647-conjugated anti-MAVS antibody (purple)and visualized using the Leica TCS SP2 laser scanning confocal microscope(Fig. 4A & B).Merged images of NS1with mitochondria and NS1 with MAVS are shown in figure 4A. Figure 4B shows the 5× enlarged images ofthe cells infected with rA2 (*top panel*) and rA2ΔNS2 (*bottom panel*) at 12 h p.i.In figure 4B, the areas marked by white circlesclearly demonstrate that NS1 (green) colocalizes with mitochondria (red)andmitochondrial MAVS proteins (purple). The bottom panel shows that NS1 colocalizes with mitochondrial MAVS independent of RSV NS2. The total merge of NS1, mitochondria and MAVS is also shown. The Pearson's and Manders' overlap coefficients derived with the JACoPImageJ tool for mitochondria and NS1 in the presence of rA2 or rA2ΔNS2 represent the average of ten individual cells and are shown in figure 4C (rA2-infected cells) and 4D (rA2ΔNS2-infected cells). M1 signifies correlation of MAVS overlapping NS1, while M2 indicates the overlap coefficient of NS1 to mitochondria. The positive Pearson's and Manders' overlap coefficients suggest colocalization of NS1 with MAVS and mitochondria, independent of NS2.

### RSV NS1 associates with MAVS in infected cells

**Figure 5:**
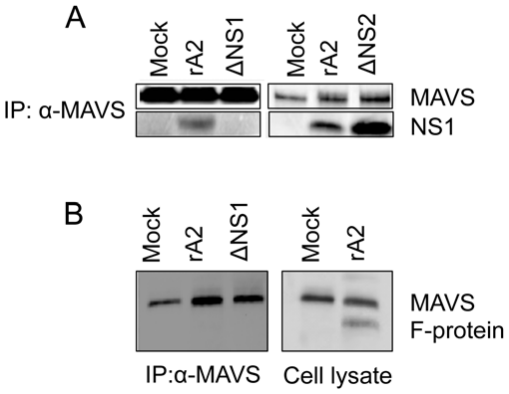
MAVS co-immunoprecipitates with NS1 in epithelial cells infected with rA2- RSV or rA2. (**A**) HEp-2 cells were eithermock-infectedor infected with rA2 or rA2ΔNS1or rA2ΔNS2 at an MOI of 1. At 24 h p.i., total cell extracts were immunoprecipitated with anti-MAVS followed by western blot analysis using anti-NS1 or anti-MAVS antibodies. (**B**) Whole cell lysates from mock or rA2 infected HEp-2 cells were immunoblotted for RSV F protein using anti RSV F antibody (Chemicon MAB8262) (*right panel*). The lysates from mock or rA2 or rA2ΔNS1 were co-immunoprecipitated with anti-MAVS antibody and probed for MAVS and RSV F protein (*left panel*).

HEp-2 cells were mock-infected or infected at an MOI of 1 with either rA2or rA2 ΔNS1 orrA2 ΔNS2 and at 24 h post-infection (p.i.), total cell lysates were immunoprecipitated with anti-MAVS antibody and analyzed by western blotting with anti-NS1 antiserum. At 24 h p.i., NS1 was detected in the anti-MAVS precipitates from lysates of rA2- and rA2 ΔNS2-infected cells, but not mock- or rA2 ΔNS1-infected cells (Fig. 5A). Whole cell lysates from mock- or rA2-infected cells at 24 h p.i were immunoblotted using anti RSV-F antibody (ChemiconMAB8262) (Fig. 5B, *rightpanel*). The specificity of NS1-MAVS interaction is shown when the same lysates (mock- or rA2- or rA2 ΔNS1-infected cells) 24 hp.i. were immunoprecipitated with anti-MAVS antibody and probed for MAVS and RSV F protein (Fig. 5B, *left panel*).

### NS1 protein binds to MAVS and colocalizes to mitochondria in transfected cells

**Figure 6:**
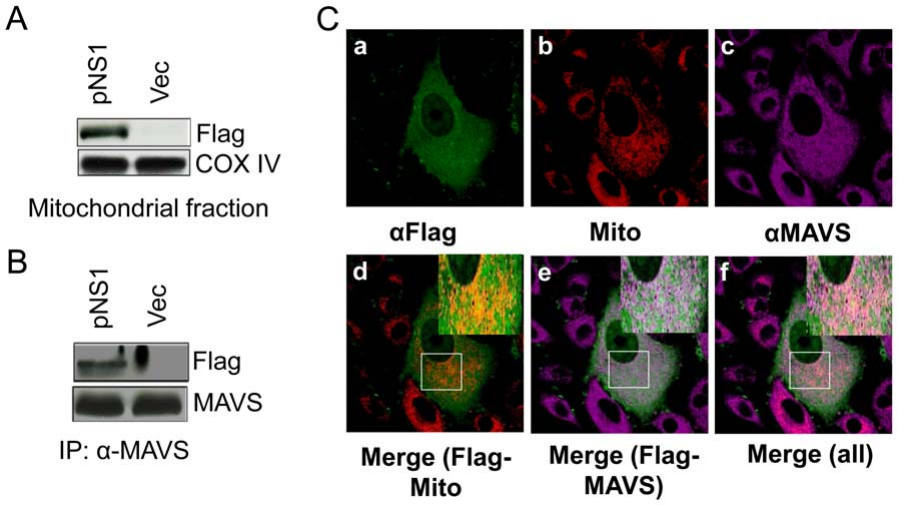
RSV NS1 is present on the mitochondria and co-immunoprecipitates with MAVS in NS1-transfected cells. (**A**). A549 cells were transfected with pNS1-Flag (NS1) or pFlag (Vec). Mitochondria were isolated and the proteins were analyzed by western blot for Flag and Cox-IV (**B**). Total cell extracts were immunoprecipitated with anti-MAVS antibody and analyzed by western blotting with anti-Flag and anti-MAVS antibodies. (**C**). A549 cells transfected with pNS1-Flag were stained for Flag (green, *a*), mitochondria (CMXRos, red, *b*), and MAVS (purple, *c*) and visualized using the Leica TCS SP2 laser scanning confocal microscope. The squares in*d*and *e* show co-localization of NS1 with mitochondria and MAVS respectively, and the box in *f* shows co-localization of NS1, MAVS and mitochondria. The insets in *d,e*, and *f* show the enlargement of the indicated areas.

A549 cells were transfected with either pNS1-Flag plasmid or pFlag plasmid. Mitochondrial proteins were separated by SDS-PAGE and blots were analyzed using rabbit anti-Flag antibody and anti-COX-IV which acts as a loading control for mitochondrial fractions. Figure 6A demonstrates that NS1 is present in the mitochondria. Immunoprecipitation of total cell extracts of the transfected cells using anti-MAVS antibody and immunoblotting for Flag showed that MAVS and Flag-NS1 are associated (Fig. 6B). Further, A549 cells were transfected with pNS1-Flag, stained for mitochondria (red, CMXRos), Flag (green), MAVS (purple), and with a nuclear stain (blue) and visualized using the Leica TCS SP2 laser scanning confocal microscope (Fig. 6C). In Figure 6C, images *a–c* showA549 cells 24 h after transfection stained for mitochondria (red, CMXRos), Flag (green, Alexa Fluor 488) and MAVS (purple, Alexa Fluor 647), respectively. Figure 6C (*d* and *e*) shows the merges between red and green and green and purple respectively, while panel *f* is the total merge between red, green, and purple. The colocalization of mitochondria and NS1-Flag is shown as yellow (marked by a square, *d*) and colocalization of MAVS and NS1-Flag is shown as white (marked by a square, *e*).

### NS1 protein inhibits RIG-I from binding to MAVS in RSV-infected cells and NS1-transfected cells

**Figure 7:**
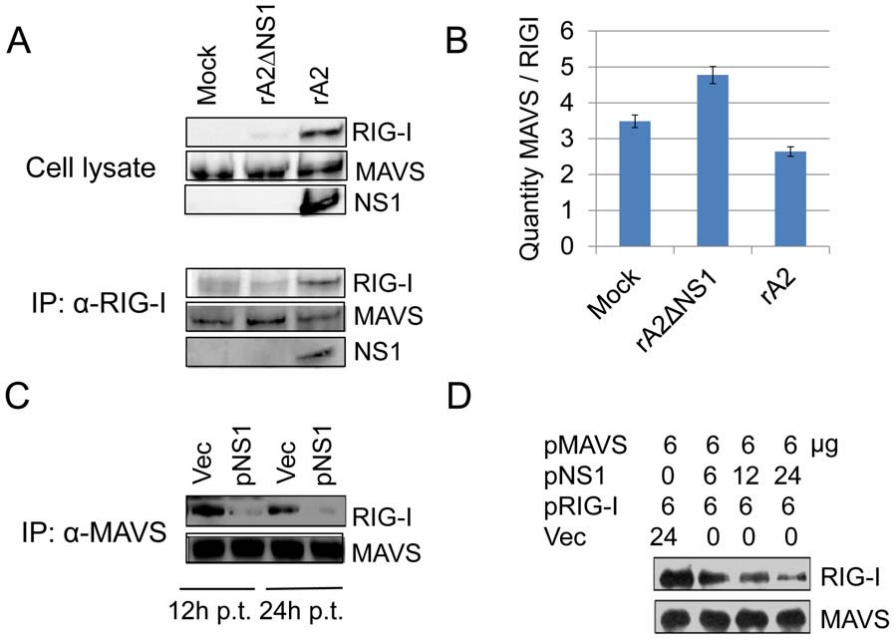
NS1 protein prevents RIG-I from binding to MAVS. (**A**) A549 cells were mock-infected or infected with rA2 or rA2ΔNS1 (MOI = 1). Whole-cell lysates were subjected to western blot analysis with anti-RIG-I, anti-MAVS, and anti-NS1antibodies (*top panel*). The same lysates were immunoprecipitated with anti-RIG-I antibody and the protein complexes were immunoblotted with the indicated antibodies (*bottom panel*). (**B**)The bar graph represents the densitometric quantification of the band intensities as the ratio of MAVS to RIG-I. (**C**) A549 cells were transfected with the indicated plasmids (12 µg of either pVAX or pNS1) and incubated with 0.2 ng/ml poly (I:C). Cytoplasmic extracts from cells 12 and 24 h after transfection were immunoprecipitated with anti-MAVS and immunoblotted with anti-RIG-I antibody. (**D**) Increasing amounts of pNS1 were co-transfected with constant amounts (6 µg) of pMAVS and pRIG-I, along with 0.2 ng/ml poly (I:C). Competitive inhibition of the interaction of MAVS with RIG-I was tested using IP, as described in (**C**). Transfection with pVAX was used to keep the total amount of transfected DNA constant.

A549 cells were infected with rA2 or rA2ΔNS1 (MOI = 1). The cells were harvested 24 h after infection and whole cell lysates were western blotted for RIG-I, MAVS and NS1. Results (Fig. 7A, *top panel*) show that rA2-infected cells induced higher expression of RIG-I compared to mock- or rA2ΔNS1-infected cells. The same lysates were immunoprecipitated with anti-RIG-I antibody and the immunocomplexes were western blotted using anti-RIG-I, anti-MAVS and anti-NS1 antibodies (Fig. 7A, *bottom panel*). Results show that at 24 h p.i., immunoprecipitation of RIG-I in rA2-infected cells pulled down decreased amounts of MAVS as compared to mock or rA2 ΔNS1 infections (Fig. 7A). Furthermore, densitometric quantification (Fig. 7B) of the band intensities of RIG-I and MAVS of the immunoprecipitation blot (Fig. 7A, *bottom panel*) show that the MAVS to RIG-I ratio was significantly decreased in the presence of NS1.

To further assess the activity of NS1 in inhibiting the interaction between RIG-I and MAVS, A549 cells were transfected with pVAX (vec) or pNS1 plasmids (2 µg/million cells) along with 0.2 ng/ml poly (I:C) as an activator of interferon responses (Fig. 7C). Figure 7C shows that NS1 expression in transfected cells disrupts the interaction between MAVS and RIG-I. The cells were harvested at 12 and 24 h after transfection and whole cell lysates were immunoprecipitated with anti-MAVS and immunoblotted with anti-RIG-I and anti-MAVS antibodies. At both time points, co-immunoprecipitation assays showed that transfection of pNS1 inhibits the association of RIG-I and MAVS in contrast to the vector control (Fig. 7C). Increasing amounts of pNS1 were cotransfected with constant amounts of pMAVS and pRIG-I, along with 0.2 ng/ml poly(I:C). Competitive inhibition of interaction of MAVS with RIG-I was determined by immunoprecipitation/immunoblotting as described in Figure 7D. Transfection with vector pVAX was used as control and to maintain a constant amount of transfected DNA. With increasing amounts of NS1 in the transfected cells, the level of interaction between MAVS and RIG-I decreases (Fig. 7D).

## Discussion

In influenza virus infection, the viral RNA is detected by RIG-I, which then interacts with MAVS leading to the induction of NF-kB-responsive genes and type-I IFN production [30], [31]. It was further demonstrated that the influenza virus employs its NS1 protein to subvert the RIG-pathway through MAVS [32], [33]. Similarly, the RSV NS2 protein binds to RIG-I, which in turn prevents its interaction with MAVS and affects downstream signaling and IFN production.A major finding of this study is that RSV NS1 protein associates with mitochondria, specifically with the MAVS protein. This binding of NS1 interferes with RIG-I-MAVS interaction, which is important for the production of IFN-β, without significantly affecting expression of either RIG-I or MAVS. In this report, we have substantiated this statement with three lines of evidence.

First, we have shown by live-cell imaging that in cells infected with rgRSV, the green fluorescent protein, which is among the first to be translated,is found in the vicinity of mitochondria that exist in the nuclear periphery. This is consistent with previous reports of localization of polio virus in perinuclear membrane complexes in Hela cells [34]. Second, wevisualized the localization of His_6_-NS1 in liveRSV-infected epithelial cells using a nickel-conjugated fluorophore and confocal microscopy.While such an approach has been used in vitro, itsin vivo use in live infected cells is innovative. Our data show that NS1 localizes to the cytoplasm near mitochondria.

In addition to our studies using the chemical labeling method for visualizing hexahistidine-tagged proteins, standard electron microscopy, and immunofluorescence wereused to determine the location of NS1 in RSV-infected cells. With the advancement of electron and confocal microscopy, subcellular localization can be observed under stable conditions in fixed or living mammalian cells; however, chemical fixation has the potential to cause artifacts. We demonstrated the application of three different microscopy techniques for detecting colocalization of RSV NS1 on the mitochondria in NS1-transfected and RSV-infected epithelial cells. The standard method of immunofluorescence is frequently used in colocalization studies and to avoid nonspecific binding and the need for potentially cross-reactive secondary antibodies, we used Zenon labeling kits. We first observed colocalization of NS1 at 16 hp.i. on CMXRos red-stained mitochondria, then we showed that NS1 colocalized with mitochondria at 10 h p.i. using a novel chemical labeling approach. We confirmed the specificity of the polyclonal anti-NS1 antibody in immunofluorescence by performing TEM analysis using Ni^2+^-NTA-nanogold. In addition, we showed co-immunoprecipitation of NS1 with MAVS, an integral mitochondrial outer membrane protein in rA2-infected cells.

Confocal and immuno-electron microscopy showed co-localization of NS1 and MAVS on mitochondria of RSV-infected cells and NS1-transfected cells. The lack of any difference in this regard between cells infected with rA2 or NS2-deficient RSV showed that NS1 binding to MAVS is not affected by NS2. To demonstrate direct interaction between NS1 and MAVS, we immunoprecipitated whole cell lysates from either mock- orrA2- or NS1- or NS2-deficient RSV-infected cells with anti-MAVS antibody, and NS1 was found to co-IP with MAVS in both rA2- and NS2-deficient RSV. Also, no other protein (RSV F) was found to interact with MAVS, showing the specificity of interaction between MAVS and NS1. At the same time in NS1-transfected cells, mitochondrial fractions showed the presence of NS1 that co-IP'd with MAVS.

We further investigated the interaction between RIG-I and MAVS in the presence of NS1. When whole cell lysates were immunoblotted, RIG-I expression was found to begreater in rA2-infected cellsthan in mock- or rA2ΔNS1-infected cells,which is consistent with previous findings [35]. When the same lysates were immunoprecipitated with anti-RIG-I antibody, MAVS and the NS1 complex were pulled down along with RIG-I. Our results demonstrate that infection of cells with rA2 ΔNS1 does not appear to affect the interaction of RIG-I with MAVS, in contrast to infection by rA2, suggesting that NS1 interference with the interaction between RIG-I and MAVS may be an important factor in RSV infection. The degree of inhibition of the antiviral response is dependent on NS1 dose. The dose-dependent regulation of RIG-I/MAVS interaction is clearly demonstrated in NS1-transfected cells. Unlike RSV NS2, which was previously observed to bind to RIG-I for inhibition of IFN-β, NS1disrupts the RIG-I/MAVS signaling through binding of MAVS, which could lead todiminished antiviral responses as our lab has previously reported [12], [23]. In contrast to Swedan et al., we utilized immunoprecipitation enrichment to observe changes in abundance of the RIG-I/MAVS complex and were not measuring total protein or RNA expression [36].While the precise mechanism of RSV NS1 inhibition of RIG-I/MAVS interaction remains unknown, clearly this differs from the hepatitis C virus (HCV) NS3-4A, a serine protease of HCV known to attenuate IFN-β production by cleaving MAVS [37].

Collectively, our evidence supports the hypothesis that RSV NS1 interferes with the antiviral signaling pathway by binding to the mitochondrial protein MAVS during early infection and disrupting the MAVS/RIG-I interaction that is necessary for signaling. To our knowledge, this is the first report that demonstrates the mitochondrial localization of NS1and our data support a critical role for NS1 in the initial stage of infection with RSV. Taken together, these data demonstrate that RSV targets the mitochondria in cells by producing a nonstructural NS1 protein that binds to MAVS and negatively regulates the RIG-I-mediated antiviral host defense responses such as type-I IFN production. The binding of NS1 to MAVS most likelyresults in steric hindrance that prevents RIG-I/MAVS interaction, but the detailed molecular mechanism of this inhibition of RIG-I and MAVS leading to interference with IFN-β production remains to be established.
